# A comprehensive evaluation of risk factors for mortality, infection and colonization associated with CRGNB in adult solid organ transplant recipients: a systematic review and meta-analysis

**DOI:** 10.1080/07853890.2024.2314236

**Published:** 2024-03-05

**Authors:** Siyu Gao, Xiaoli Huang, Xiaolin Zhou, Xiangcheng Dai, Jing Han, Yandong Chen, Hongliang Qiao, Yi Li, Yifan Zhou, Ting Wang, Huiqing He, Qiang Liu, Shenjie Tang

**Affiliations:** a Department of Tuberculosis, Beijing Chest Hospital, Capital Medical University, Beijing Tuberculosis and Thoracic Tumor Research Institute, Beijing, China; b Department of Infectious Diseases, Yichang Central People’s Hospital, The First College of Clinical Medical Science, China Three Gorges University, Yichang, China; c Department of Urology, Yichang Central People’s Hospital, The First College of Clinical Medical Science, China Three Gorges University, Yichang, China; d Department of Cardio-Thoracic Surgery, Yichang Central People’s Hospital, The First College of Clinical Medical Science, China Three Gorges University, Yichang, China; e National Health Commission of the People’s Republic of China, Yichang, China

**Keywords:** Solid organ transplant, CRGNB, infection, colonization, mortality

## Abstract

**Background:**

The burden of carbapenem-resistant gram-negative bacteria (CRGNB) among solid organ transplant (SOT) recipients has not been systematically explored. Here, we discern the risk factors associated with CRGNB infection and colonization in SOT recipients.

**Methods:**

This study included observational studies conducted among CRGNB-infected SOT patients, which reported risk factors associated with mortality, infection or colonization. Relevant records will be searched in PubMed, Embase and Web of Science for the period from the time of database construction to 1 March 2023.

**Results:**

A total of 23 studies with 13,511 participants were included, enabling the assessment of 27 potential risk factors. The pooled prevalence of 1-year mortality among SOT recipients with CRGNB was 44.5%. Prolonged mechanical ventilation, combined transplantation, reoperation and pre-transplantation CRGNB colonization are salient contributors to the occurrence of CRGNB infections in SOT recipients. Renal replacement therapy, post-LT CRGNB colonization, pre-LT liver disease and model for end-stage liver disease score increased the risk of infection. Re-transplantation, carbapenem use before transplantation and ureteral stent utilization increaesd risk of CRGNB colonization.

**Conclusion:**

Our study demonstrated that SOT recipients with CRGNB infections had a higher mortality risk. Invasive procedure may be the main factor contribute to CRGNB infection.

## Introduction

The emergence of carbapenem-resistant gram-negative bacteria (CRGNB), including carbapenem-resistant *Klebsiella pneumoniae* (CRKP), carbapenem-resistant *Acinetobacter baumannii* (CRAB), carbapenem-resistant *Pseudomonas aeruginosa* (CRPA) and others, has become a global public health emergency with few therapeutic options, especially among solid organ transplant (SOT) recipients [1]. The isolation of CRGNB has become a growing concern. SOT itself has also been independently associated with the development of carbapenem-resistant Enterobacteriaceae (CRE) acquisition [2,3]. However, to date, the relationship between risk factors and CRGNB in SOT recipients remains unclear due to the small number of scattered cases and inconsistent publications with varying quality across larger studies. Moreover, many studies have reported that the presence of CRGNB infection is associated with a high risk of death but without a relatively accurate mortality in SOT recipients [4]. The objective of this systematic review and meta-analysis was to examine this relationship within the context of CRGNB endemicity and to clarify the risk factors for mortality, colonization and infection in SOT recipients.

## Materials and methods

This study was conducted in accordance with the Preferred Reporting Items for Systematic Reviews and Meta-Analyses (PRISMA) guidelines [5]. The review protocol was prospectively registered with the National Institute for Health Research PROSPERO system (http://www.crd.york.ac.uk/prospero; Registration No. CRD42022306168).

### Search strategy

A systematic electronic search of English-written articles was performed to identify all relevant studies published up to 1 March 2023, in PubMed, Embase and Web of Science. The following search terms were used: transplant* AND infection AND ‘carbapenem resistant’ OR ‘imipenem resistant’ OR ‘meropenem resistant’ OR ‘ertapenem resistant’ OR ‘doripenem resistant’. The references of the selected articles and relevant review articles were screened for potential misses.

### Eligibility criteria

Eligible studies were case–control or cohort studies that investigated the association of CRGNB colonization, infection and mortality, with available data including hazard ratios (HRs), risk ratios (RRs), odds ratios (ORs) or death rates. Studies were excluded if they were (1) duplicated studies; (2) reviews, reports or meeting abstracts; (3) studies that did not distinguish between infection and colonization; (4) studies that did not provide sufficient data for 95% confidence intervals (CIs) and HRs, RRs or ORs; (5) studies that included only CRGNB cases in the absence of a comparison group comprised of SOT recipients without CRGNB and (6) studies from paediatric patients. When two or more studies from the same institution and the same author assessed the same risk factors, the study with the longest study period was selected for analyses [6]. Both records were included if they assessed the different CRGNB [7,8]. Moreover, to control for any confounding effects in each study, only studies that performed multivariable analysis were included in the meta-analysis of risk factors.

### Definition

Colonization was defined as the isolation of CRGNB from rectal swab from asymptomatic patients during admission. Collected rectal swab specimens were cultured and species identification was performed in the presence of CRGNB growth. Isolates identified as CRGNB were ascertained as CRGNB colonization. Positive surveillance cultures of clinical samples (including urine, respiratory samples and other skin cultures) in the absence of symptoms and signs of infection also were considered for colonization status. Rectal screening to identify CRGNB carriage was usually implemented before and after surgery as well as before and after transferred to intensive care unit (ICU). Recipients found to be colonized with CRGNB by observation performed during the hospital stay or at the time of transplantation were ascertained as CRGNB carriers at transplantation, and conversely those found to be colonized afterwards were ascertained as having acquired CRGNB carriage post-transplantation. Colonization or infection during ICU stay is the same. The infection was ascertained by integrating positive cultures of different samples with corresponding positive clinical manifestations. The occurrence of infection was defined as the presence of a bacterial pathogen in clinical samples (e.g. blood, respiratory secretions, ascites, pleural fluid, cerebrospinal fluid or surgical sites) in combination with clinical signs and symptoms of infection. We defined CRGNB as a strain of gram-negative bacteria resistant to at least one of the carbapenems with a minimum inhibitory concentration according to the standards of the Clinical and Laboratory Standards Institute (CLSI) or The European Committee on Antimicrobial Susceptibility Testing (EUCAST) [9,10]. The mortality was defined as deaths attributable to any cause.

### Data extraction

Two researchers independently and blindly completed the literature screening, data extraction and quality evaluation. They browsed the title and abstract of the document to exclude irrelevant literature. They then extracted the data and documented the following details in standardized tables: first author, year and country, period, research type, study design, sample size, transplant type, infection type, identification method, risk factors, mortality and results of other events of interest. If the results differed, a third researcher would be consulted for judgement.

### Statistical analysis and assessment of risk bias

For single-group data for mortality, pooled point mortality estimates regarding the relatively short observational period (30-day to 1-year mortality) along with 95%CIs for the included studies were calculated. Random-effects models were used to address expected heterogeneity.

When at least two studies analysed a potential risk factor, and the definition of such a factor was consistent across studies, we performed a meta-analysis of risk factors. Only the effect estimates (HRs, RRs or ORs) and 95%CIs of the multivariate analysis models were extracted from original studies. To estimate heterogeneity, *P* values for heterogeneity and *I^2^* statistics were calculated. The random-effects model was used to combine the results when heterogeneity was present among the studies (*I*^2^ > 50% or *P* < 0.05). Otherwise, a fixed effects model was applied. Egger’s and Begg’s tests were used to assess publication bias statistically. For any variable presenting with large heterogeneity, a sensitivity analysis that excluded outlier studies was conducted to investigate the potential origin of heterogeneity. *P* < 0.05 was considered statistically significant for all included studies. The quality of each study was assessed using the Newcastle-Ottawa Quality Scale (NOS) [7]. This scale assesses each study using three categories: (1) representativeness of the subjects, (2) comparability between the study groups and (3) ascertainment of the exposure or outcome of interest for case–control and cohort studies. Studies with a total score >6 and <4 were considered high and low quality, respectively. Statistical analyses were performed using the Stata software (version 15.0; Stata Corporation, College Station, TX, USA) and RevMan (Review Manager, version 4.3).

## Result

### Study selection

**Figure 1.**
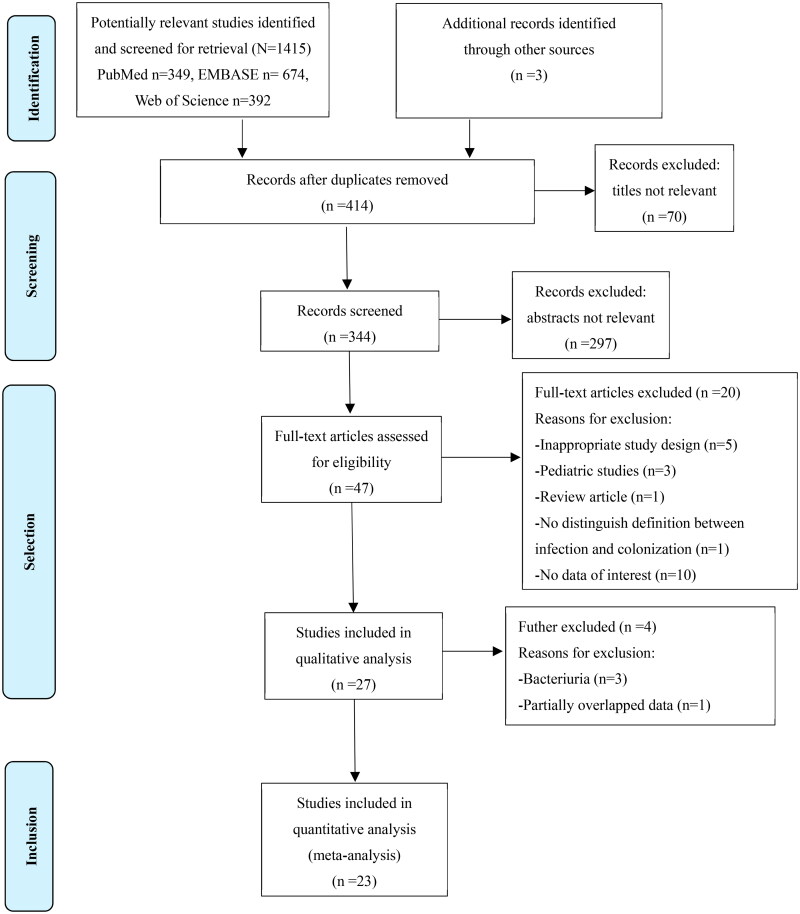
Flow diagram for literature search and study selection.

The flowchart of study selection is shown in Figure 1. A total of 4556 records were retrieved from the electronic databases and reference lists of retrieved studies and relevant systematic reviews. After removing duplicate studies and a detailed evaluation of titles and abstracts, 47 studies were identified for full-text screening. A total of 27 studies were further screened in detail. Three studies of bacteriuria were excluded because they were difficult to classify as infection or colonization and lacked consistent sampling methods. Two studies conducted the same research on the same group of patients over a certain period, and one of them with a short total study time, was excluded. Ultimately, 23 studies met the inclusion criteria. Six studies were from the same author but comprised consecutive nonoverlapping cohorts [6,7,11–14].

### Study characteristics

Table 1 presents the basic characteristics of the studies. In total, 13,511 participants were included in the analysis. The sample sizes of the studies ranged from 78 to 3005. These included studies were published from 2005 to 2020 in different countries, including China [8,15,20,29], Brazil [6,7,11–14,21,24], Turkey [16], Korea [17,18], Italy [19,26–28,30] and the United States [22,23,25]. Of the 23 studies, 6 were retrospective cohort studies, 5 were prospective cohort studies and 12 were case–control studies. Approximately half of the studies (*n* = 12, 52.2%) recruited liver transplantation (LT) recipients, seven studied kidney transplantation (KT; *n* = 7, 30.4%) and the rest (*n* = 4, 17.4%) from SOT (the SOT included heart, lung, liver, kidney, pancreas and combined organ transplants). CRKP was the most common isolate (*n* = 11, 47.8%). All studies were considered high-quality research after assessment via NOS. The outcomes of the NOS are shown in Supplementary Table S1.

**Table 1.** The characteristics of the 23 included studies.

Study	Country	Study design	Period	Organ	Bacterial species	Population size	Case/control^a^, *n*	Follow-up	NOS points
Chen et al. [15]	China	Case–control	2017–2019	LT	CRE	387	Infection, 26/361	30 days	8
Freire et al. [11]^b^	Brazil	Case–control	2009–2014	KT	CRE	78	Colonization, 26/52	4243 days	8
Cinar et al. [16]	Turkey	Case–control	2014–2018	LT	CPE	142	Infection, 37/105	NA	7
Freire et al. [6]^b^	Brazil	Case–control	2010–2019	KT	CRE	977	Colonization, 323/646; infection, 107/206	NA	8
Kim et al. [17]	Korea	Case–control	2008–2015	LT	CRAB	393	Infection, 14/379	2620 days	7
Freire et al. [12]^b^	Brazil	Case–control	2019–2020	KT	CRPA	151	Colonization, 37/111	NA	8
Lee et al. [18]	Korea	Case–control	2015–2016	SOT	CPE	309	Colonization, 37/100	350 days	7
Giannella et al. [19]	Italy	Prospective cohort	2010–2017	LT	CPE	553	Colonization, 147/406; infection, 57/496	NA	8
Freire et al. [7]^b^	Brazil	Prospective cohort	2010–2014	LT	CRE	401	Colonization, 119/206; infection, 54/324	60 days	7
Wu et al. [8]	China	Retrospective cohort	2013–2020	LT, KT	CRGNB	1452	Mortality, 23/130	90 days	8
Freire et al. [13]^b^	Brazil	Prospective cohort	2009–2011	LT	CRAB	202	Colonization, 81/91; infection, 56/138	60 days	7
Zhang et al. [20]^b^	China	Retrospective cohort	2017–2019	KT	CRKP	419	Infection, 43/107; mortality, 23/127	90 days; 1 year	9
Pagani et al. [21]	Italy	Case–control	2011–2014	SOT	CRKP	920	Infection, 35/793	1 year	8
Hong Nguyen et al. [22]	USA	Prospective cohort	2015–2017	SOT	CRE	171	Infection, 10/145	5.3 years	8
Freire et al. [14]^b^	Brazil	Retrospective cohort	2009–2013	KT	CRKP	1111	Infection, 25/1076	1288 days	7
Kalpoe et al. [23]	USA	Retrospective cohort	2005–2006	LT	CRKP	175	Infection, 14/161	1 year	8
Taminato et al. [24]	Brazil	Case–control	2011–2016	KT	CRKP	3005	Infection, 45/90	NA	8
Pereira et al. [25]^b^	USA	Retrospective cohort	2010–2013	LT	CRKP	305	Infection, 20/284	1 year	7
Mazza et al. [26]	Italy	Retrospective cohort	2012–2015	LT	CRKP	310	Infection, 8/302	180 days	7
Giannella et al. [27]	Italy	Prospective cohort	2010–2013	LT	CRKP	237	Infection, 20/217	180 days	7
Varotti et al. [28]	Italy	Case–control	2010–2014	KT	CRKP	234	Infection, 26/56	>180 days	7
Wu et al. [29]	China	Retrospective cohort	2012–2019	SOT	CRKP	1249	Mortality, 20/48	90 days	8
Barchiesi et al. [30]^b^	Italy	Case–control	2005–2014	LT	CRKP	330	Mortality, 27/61	180 days	7

Abbreviations: LT, liver transplantation; KT, kidney transplantation; SOT, solid organ transplantation; CRE, carbapenem-resistant Enterobacteriaceae; CRAB, carbapenem-resistant *Acinetobacter baumannii*; CRPA, carbapenem-resistant *Pseudomonas aeruginosa*; CRGNB, carbapenem-resistant gram-negative bacteria; CRKP, carbapenem-resistant *Klebsiella pneumoniae*; NA, not available.

^a^Case/control showed available data including infected and uninfected groups, colonized and not-colonized groups and mortality and survival groups.

^b^Studies were conducted in regions of CRGNB endemicity.

### Risk factors for mortality of SOT recipients with CRGNB infections

**Figure 2.**
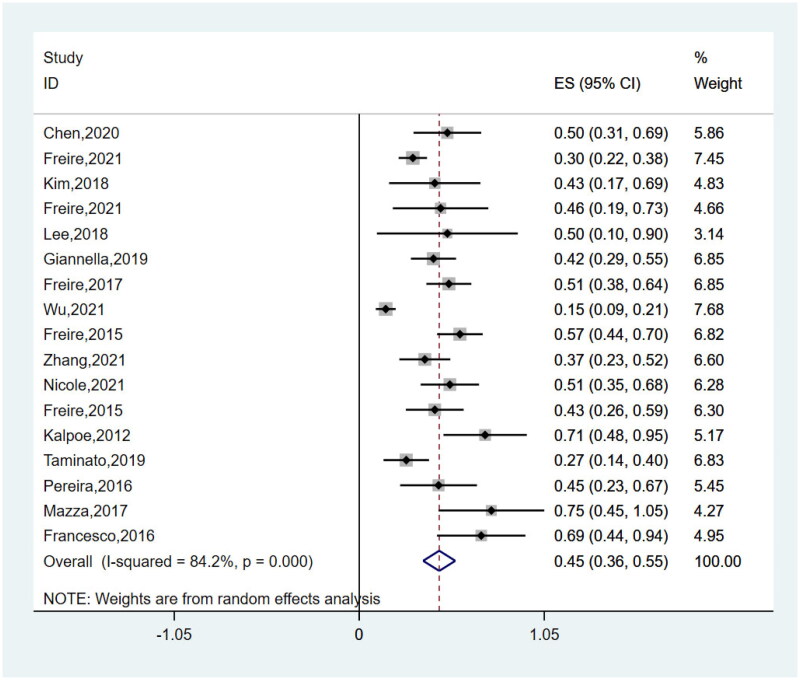
Forest plots for pooled mortality (1-year) of SOT recipients with CRGNB infections in included studies.

Post-transplantation CRGNB infections (OR 4.58, 95%CI 3.14–6.70) and reoperation (OR 2.19, 95%CI 1.61–2.98) were found to be significantly associated with mortality in SOT recipients (*P* < 0.00001). All the risk factors for mortality are summarized in Supplementary Table S2. The pooled mortality (1-year) of SOT recipients with CRGNB infections was 44.5% (95%CI 0.36–0.53%) in these studies (Figure 2). *I*^2^ for a heterogeneity of 83.2% revealed high heterogeneity. The analysis included 722 participants from 18 studies. Interestingly, subgroup analyses concerning the date of pooled mortality assessment (Supplementary Figure S1) indicated the same mortality between 1-year and 180-day (44.2%, 95%CI 0.33–0.57%), and a small reduction in 90-day mortality (39.5%, 95%CI 0.24–0.55%). Moreover, subgroup analyses of 1-year mortality according type of transplantation showed LT recipients exhibit a higher mortality rate (49.6%, 95%CI 34.3–64.9%), in contrast to KT (30.3%, 95%CI 16.6–45.9%). Supplementary Figure S2illustrates forest plots for the risk factors of mortality. No significant differences were observed between subgroups (Supplementary Figure S3: subgroup analyses of risk factors for mortality).

### Risk factors for CRGNB infections in SOT recipients

In the meta-analysis of the post-transplant occurrence of CRGNB infections, the pooled results demonstrated that these factors increase the risk of CRGNB infections, including prolonged mechanical ventilation (OR 2.99, 95%CI 1.93–4.63), combined transplantation (OR 3.46, 95%CI 2.12–5.65), re-operation (OR 2.09, 95%CI 1.42–3.09), pre-transplantation CRGNB colonization (OR 12.91, 95%CI 5.23–31.88) and the mean length of post-transplantation ICU stay (OR 1.13, 95%CI 1.11–1.15; Table 2). However, cold ischemia time, age and delayed graft function were not significantly associated with CRGNB infections (OR 1.01, 95%CI, 0.99–1.04; OR 1.04, 95%CI, 1.00–1.08; OR 2.09, 95%CI 0.68–6.46). In addition, other variables for LT, such as renal replacement therapy (OR 3.05, 95%CI 2.08–4.50), post-LT CRGNB colonization (OR 8.58, 95%CI 4.08–18.04), pre-LT liver disease (OR 4.14, 95%CI 2.29–7.46), Model for end-stage liver disease (MELD) score (OR 1.04, 95%CI 1.02–1.06) and biliary complications (OR 4.92, 95%CI 2.16–11.23) also considerably increased infection risk (Supplementary Figure S4 illustrates the forest plots for risk factors of infection). Subgroup analyses showed differences in the type of organ and bacteria observed in pre-LT CRGNB acquisition (Supplementary Figure S5: subgroup analyses of risk factors for infection).

**Table 2.** Risk factors for CRGNB infections after SOT recipients.

	Number of studies	Heterogeneity			OR [95%CI]	*Z*	*P*	Effect model	Egger’s test, *P* > |*t*|	Begg’s test
		*χ* ^2^	*P*	*I* ^2^						
SOT										
Prolonged mechanical ventilation	5	1.87	0.76	0%	2.99 [1.93, 4.63]	4.91	<0.00001	Fixed	0.755	0.806
Combined transplantation	4	1.61	0.66	0%	3.46 [2.12, 5.65]	4.98	<0.00001	Fixed	0.583	0.734
Reoperation	5	5.76	0.22	31%	2.09 [1.42, 3.09]	3.72	0.0002	Fixed	0.532	0.221
Pre-transplantation CRGNB colonization	5	13.52	0.009	70%	12.91 [5.23, 31.88]	5.55	<0.00001	Random	0.425	0.462
Delayed graft function	3	5.03	0.08	60%	2.09 [0.68, 6.46]	1.28	0.2	Random	0.694	1
Mean length of post-transplantation ICU stay	2	1.63	0.2	39%	1.13 [1.11, 1.15]	13.54	<0.00001	Fixed	NA	1
LT										
Renal replacement therapy	6	4.1	0.53	0%	3.06 [2.08, 4.50]	5.67	<0.00001	Fixed	0.967	1
Post-LT CRGNB colonization	4	7.8	0.05	62%	8.58 [4.08, 18.04]	5.67	<0.00001	Random	0.367	0.734
Pre-LT liver disease^a^	4	1.84	0.61	0%	4.14 [2.29, 7.46]	4.72	<0.00001	Fixed	0.361	0.308
MELD score	6	7.04	0.22	29%	1.04 [1.02, 1.06]	3.29	0.001	Fixed	0.224	0.707
Cold ischaemia time	2	8.1	0.004	88%	1.01 [0.99, 1.04]	0.96	0.34	Random	NA	1
Biliary complication	2	0.27	0.6	0%	4.92 [2.16, 11.23]	3.78	0.0002	Fixed	NA	1
KT										
Age	2	1.32	0.25	24%	1.04 [1.00, 1.08]	2.07	0.04	Fixed	NA	1

Abbreviations: ICU, intensive care unit; MELD, model for end-stage liver disease.

^a^Pre-LT liver disease includes histological recurrence of hepatitis C virus infection, fulminant hepatitis, alcoholic liver disease and hepatocellular carcinoma.

### Risk factors for CRGNB colonization in SOT recipients

Next, we investigated the risk factors for CRGNB colonization in SOT recipients (Table 3). The presence of re-transplantation (OR 10.43, 95%CI 3.45–31.33) and carbapenem use before transplantation (OR 3.78, 95%CI 1.28–11.14) were associated with a significantly increased risk of CRGNB colonization. However, hospital stay was not found to be significant (OR 4.79, 95%CI 0.69–33.44, *P* > 0.05). The data provided for three studies [6,11,12] on KT noted a positive association between ureteral stent use and CRGNB colonization (*P* < 0.0001), and the pooled OR was 1.94 (95%CI 1.40–2.68). Age (OR 1.20, 95%CI 0.86–1.68) and low median lymphocyte count in the last 3 months (OR 0.43, 95%CI 0.02–10.95) were not found to be significant (*P* > 0.05; Supplementary Figure S6 illustrates the forest plots for risk factors of colonization). Nearly no significant differences were observed between subgroups (Supplementary Figure S7: subgroup analyses of risk factors for colonization).

**Table 3.** Risk factors for CRGNB colonization after SOT recipients.

	Number of studies	Heterogeneity			OR [95%CI]	*Z*	*P*	Effect model	Egger’s test, *P* > |*t*|	Begg’s test
		*χ* ^2^	*P*	*I* ^2^						
SOT										
Re-transplantation	3	0.49	0.78	0%	10.43[3.47, 31.33]	4.18	<0.0001	Fixed	0.655	1
Previous carbapenem use	3	5.67	0.06	65%	3.78 [1.28, 11.14]	2.41	0.02	Random	0.733	1
Hospital stay	3	16.21	0.0003	88%	4.79 [0.69, 33.44]	1.58	0.11	Random	0.02	0.296
KT										
Age	2	3.8	0.05	74%	1.20 [0.86, 1.68]	1.05	0.29	Random	NA	1
Ureteral stent use	2	1.68	0.19	41%	1.94 [1.40, 2.68]	4.02	<0.0001	Fixed	NA	1
Low median lymphocyte count in the last 3 months	2	10.75	0.001	91%	0.43 [0.02, 10.95]	0.51	0.61	Random	NA	1

## Discussion

Considerable evidence confirms the detrimental impact of CRGNB infections on transplant recipients [1], as well as the non-negligible role of CRGNB infections, with estimates of mortality rates up to 44.5% within one year, as the pre-eminent risk factor leading to death. Using this substantial SOT population, we identified the risk factors associated with both CRGNB colonization and infection. It is important to note that the relatively infrequent incidence of CRGNB infections has contributed to the paucity of studies assessing risk factors. To the best of our knowledge, this is the first systematic review and meta-analysis to identify risk factors for CRGNB burden in SOT recipients.

There are almost no definite data on mortality. In our study, the combined analysis involving mortality studies showed that the 1-year mortality rate of SOT recipients with CRGNB infection was 44.5%. The mortality rates ranged from 15% to 75% in each study. Among them, the study with the lowest mortality rate came from China [8]. Chinese patients often leave the hospital before death, potentially resulting in low mortality rates [31]. In contrast, in another study, up to 75% mortality might have been exaggerated due to the small number of positive cases [26]. In the meantime, subgroup analyses of mortality showed that the mortality at 1-year and 180-day is almost consistent, which may indicate that the mortality after 180 days is no longer affected by CRGNB infections. Similar to a recent meta-analysis, Xu et al. reported a mortality rate of 43.13% among SOT patients infected with CRKP; however, their research did not explain different period of mortality [32]. The outcomes of transplant patients are often complex, and despite subgroup analyses focusing on transplanted organs, the heterogeneity persists. Contrary to our findings, the study conducted by Lu et al. reveals an increase in mortality associated with KT compared to LT and lung transplantation [33]. Nevertheless, this may illustrate, to some extent, the crucial role of CRGNB infection in mortality and outcomes.

Notably, some studies have been conducted not only in endemic transplant centres or hospitals with a certain risk of CRGNB infection, but also during periods when novel antimicrobial agents targeting the CRGNB were unavailable, which may have overestimated the actual incidence.

Whether pre- or post-transplantation, the presence of CRGNB colonization increases susceptibility to CRGNB infection. In addition, previous evidence has suggested that about half of the patients were colonized with CRKP prior to bloodstream infection [34,35]. Risk factors for post-transplantation infection are not just colonization, but other factors are crucial. Our study also found that SOT recipients with CRGNB infections had a longer post-transplantation ICU stay and were more likely to receive prolonged duration of mechanical ventilation. Transference to the ICU and protective mechanical ventilation were nothing to blame after the operation by convention. However, shortening the duration of ICU stay and minimizing the use of invasive mechanical ventilation could reduce the incidence of CRGNB infections. Solid organ transplantation and ICU stay are the most important conditions associated with a significant risk of CRE infections [36–38].

Moreover, mechanical ventilation causes cyclic changes in cardiac load and puts extra pressure on the tracheal mucosa, thereby influencing physiological defence function [39,40]. Emma et al. observed that the composition of the respiratory microbiota deviated [41]. These pathogenic bacteria, including the antibacterial drug-resistant bacteria, gradually dominate within 48–72 h subsequent to antibiotic administration [42]. Similarly, combined transplantation, biliary complications, reoperation and renal replacement therapy are associated with a significant increase in CRGNB infections. These findings collectively underscore the role of invasive procedures and surgical interventions, which tend to evoke frequent barrier injuries, thereby compromising the body’s inherent resistance mechanisms and augmenting susceptibility to CRGNB exposure.

In addition, our research shows that cold ischemia time and delayed graft function have no significant correlations with CRGNB infections. In theory, extended cold ischemia time and delayed graft function could potentially affect recipient immune function under certain circumstances, thereby potentially increasing the risk of infection. However, there is currently a lack of direct evidence to establish a definite relationship between graft status and occurrence of infections.

In a word, our study suggests that more than the graft-related aspects, it is the invasive procedures, complex operations and ensuing complications that may be the main factors in precipitating CRGNB infections.

The propensity for CRGNB colonization in SOT patients parallels the risk observed in the ICU setting, including hospitalization duration and invasive procedural interventions. Dautzenberg et al. analysed 1077 patients in the ICU and found that patients colonized with CRE had, on average a 1.79 times higher risks of death, mainly attributed to prolonged hospital stay [38].

Re-transplantation and ureteral stent use were associated with the highest risk of CRGNB colonization in our meta-analysis. In KT recipients, the augmented vulnerability to urinary tract infections and the utilization of ureteral stents after transplantation contribute to heightened colonization prospects. What makes it hard to explain is that age is a risk factor for colonization and infection, especially in KT recipients.

The use of antibiotics as a recognized risk factor merits further exploration of its potential effects on the timing and species of patient-acquired CRGNB. Regrettably, there is not enough data to perform an exhaustive analysis due to a lack of study. However, some valuable clues remain. Carbapenem use was the most common risk factor in the included studies. Prior use of piperacillin/tazobactam [13] and ciprofloxacin use [12] were considered independent risk factors, with antibiotic exposure occurring within the preceding 3 months. In a murine model of intestinal colonization, the timing of antibiotic use was critical for the effective colonization of CPE [43]. Therefore, avoiding antibiotic misuse is significant for the management of CRGNB epidemic spread, while further research is required to elucidate the optimal timing of antibiotic administration.

We summarized the time to infection according to colonization status before or after transplantation, as well as the time from colonization acquisition pre-transplant and the risk of infection (Supplementary Tables S3–S4). Interestingly, we found that patients with colonization prior to transplantation were at a higher risk of infection following organ transplantation than those who acquired colonization during the post-transplantation period. This information was reported in a few studies and analysis was not possible, but this is useful information for planning preventive strategies.

Although some infection-prevention and control interventions were implemented on some included studies, no comparative data regarding the prevalence of CRGNB colonization. These strict contact precautions are convenient and acceptable, and we still believe measures able to prevent colonization are crucial to contain CRKP spread and to reduce the potential for post-transplantation infections [44,45].

The present meta-analysis only included studies of moderate to high quality and was conducted in a strict and comprehensive process. Despite the strict approach undertaken, this study is not without limitations, which merit acknowledgement and consideration. Firstly, nearly all the studies selected for analysis were observational, primarily originating from the CRGNB epidemic area, which could have potential impacts on the final outcomes. Secondly, it has some inherent weaknesses stemming from the amalgamation of studies displaying certain heterogeneity, such as divergent geographical locales, distinct time periods, variable original disease profiles and disparate follow-up durations. Thirdly, not all the included studies evaluated the same risk factors. Consequently, the pooling of data is derived from subsets of studies, and the analysis might omit a single risk factor due to data availability constraints. Moreover, subgroup analyses were performed based on the type of bacteria and transplanted organs. Notwithstanding the absence of overt dissimilarities in these subgroup analyses, the interpretation still necessitates cautious consideration owing to the potential residual confounding factors and the inherent limitations imposed by sample sizes. The intricate interplay of factors influencing CRGNB infections and their manifestations within subgroups further accentuates the complexity of this relationship. In any case, we also look forward to providing insights into risk factors, thereby contributing to a more comprehensive understanding of this critical healthcare concern. Overall, these limitations highlight the need for well-designed studies evaluating the impact of CRGNB in SOT recipients to provide informative insights for this population.

## Conclusions

Our study identified risk factors among SOT recipients associated with CRGNB infection and colonization, and several identified factors resonate with medical interventions encountered within the ICU. It is our conviction that these risk signals contribute to a comprehensive understanding of CRGNB and have the potential to profoundly influence clinical decision-making processes, which would also help contain CRGNB spread and reduce the potential for post-transplantation infections by judicious antibiotic administration, subtractive unnecessary invasive procedure and preventive isolation strategies.

## Supplementary Material

Supplemental Material

## Data Availability

The authors confirm that the data supporting the findings of this study are available within the article and its supplementary materials.

## Abbreviations

CRGNB: carbapenem-resistant gram-negative bacteria
SOT: solid organ transplant
LT: liver transplantation
CRKP: carbapenem-resistant *Klebsiella pneumoniae*
CRAB: carbapenem-resistant *Acinetobacter baumannii*
CRPA: carbapenem-resistant *Pseudomonas aeruginosa*
CRE: carbapenem-resistant Enterobacteriaceae
PRISMA: Preferred Reporting Items for Systematic Reviews and Meta-Analyses
HRs: hazard ratios
RRs: risk ratios
ORs: odds ratios
CIs: confidence intervals
CLSI: Clinical and Laboratory Standards Institute
EUCAST: The European Committee on Antimicrobial Susceptibility Testing
NOS: Newcastle-Ottawa Quality Scale
KT: kidney transplantation
ICU: intensive care unit
MELD: model for end-stage liver disease
