# Supplementary material for: A comprehensive evaluation of risk factors for mortality, infection and colonization associated with CRGNB in adult solid organ transplant recipients: a systematic review and meta-analysis
Source: Ann Med. 2024 Mar 5;56(1):2314236. doi: 10.1080/07853890.2024.2314236 (PMC10916923; doi:10.1080/07853890.2024.2314236)

Study or Subgroup	log[Odds Ratio]	SE	Weight	Odds Ratio IV, Fixed, 95% CI
**Reoperation**				
Cinar,2019	0.6043	0.569	11.7%	1.83 [0.60, 5.58]
Freire,2015(CRAB)	1.1663	0.5063	14.7%	3.21 [1.19, 8.66]
Freire,2017	0.4121	0.2364	67.6%	1.51 [0.95, 2.40]
Zhang,2021	1.0357	0.7968	6.0%	2.82 [0.59, 13.43]
**Total (95% CI)**			**100.0%**	**1.79 [1.22, 2.62]**

Heterogeneity: Chi² = 2.17, df = 3 (P = 0.54); I² = 0%
Test for overall effect: Z = 3.00 (P = 0.003)

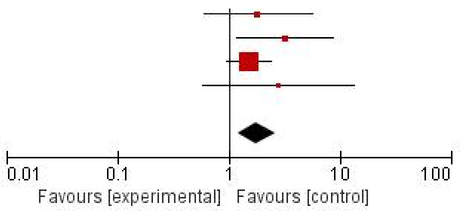

Study or Subgroup	log[Odds Ratio]	SE	Weight	Odds Ratio IV, Fixed, 95% CI
**Post-transplantation infections**				
Francesco,2016	1.716	0.7885	6.5%	5.56 [1.19, 26.09]
Freire,2015(CRAB)	0.9478	0.3497	32.9%	2.58 [1.30, 5.12]
Kalpoe,2012	1.5892	0.604	11.0%	4.90 [1.50, 16.01]
Nicole,2021	0.9207	0.5785	12.0%	2.51 [0.81, 7.80]
Pereira,2016	1.9344	0.3872	26.8%	6.92 [3.24, 14.78]
Wu,2020	3.0926	0.828	5.9%	22.03 [4.35, 111.66]
Zhang,2021	1.9097	0.949	4.5%	6.75 [1.05, 43.37]
**Total (95% CI)**			**100.0%**	**4.49 [3.03, 6.65]**

Heterogeneity: Chi² = 8.75, df = 7 (P = 0.27); I² = 20%
Test for overall effect: Z = 7.49 (P < 0.00001)

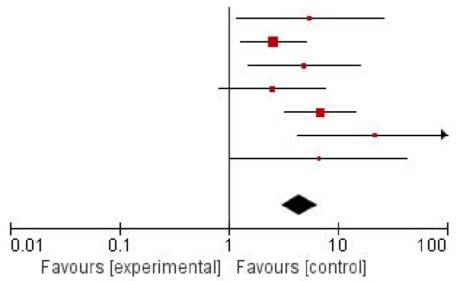

Study or Subgroup	log[Odds Ratio]	SE	Weight	Odds Ratio IV, Fixed, 95% CI
**Delayed graft function**				
Mazza,2017	1.7405	0.3275	85.7%	5.70 [3.00, 10.83]
Zhang,2021	0.674	0.8013	14.3%	1.96 [0.41, 9.44]
**Total (95% CI)**			**100.0%**	**4.89 [2.70, 8.86]**

Heterogeneity: Chi² = 1.52, df = 1 (P = 0.22); I² = 34%
Test for overall effect: Z = 5.24 (P < 0.00001)

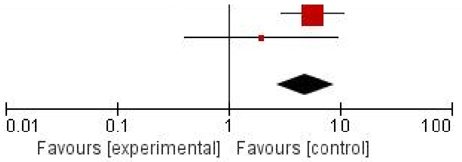

Study or Subgroup	log[Odds Ratio]	SE	Weight	Odds Ratio IV, Fixed, 95% CI
**Mechanical ventilation**				
Wu,2021	2.0015	0.5163	76.2%	7.40 [2.69, 20.36]
Zhang,2021	2.6304	0.9249	23.8%	13.88 [2.27, 85.04]
**Total (95% CI)**			**100.0%**	**8.59 [3.55, 20.79]**

Heterogeneity: Chi² = 0.35, df = 1 (P = 0.55); I² = 0%
Test for overall effect: Z = 4.77 (P < 0.00001)

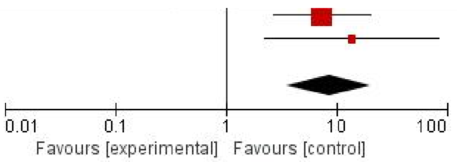

Study or Subgroup	log[Odds Ratio]	SE	Weight	Odds Ratio IV, Fixed, 95% CI
**Septic shock**				
Wu,2020	3.2992	1.3719	11.1%	27.09 [1.84, 398.65]
Wu,2021	2.0028	0.4857	88.9%	7.41 [2.86, 19.20]
**Total (95% CI)**			**100.0%**	**8.56 [3.49, 21.00]**

Heterogeneity: Chi² = 0.79, df = 1 (P = 0.37); I² = 0%
Test for overall effect: Z = 4.69 (P < 0.00001)

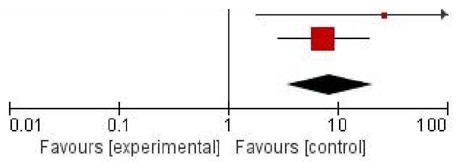

Study or Subgroup	log[Odds Ratio]	SE	Weight	Odds Ratio IV, Fixed, 95% CI
**Fulminant hepatitis**				
Freire,2015(CRAB)	1.0647	0.5382	21.8%	2.90 [1.01, 8.33]
Freire,2017	0.7227	0.2843	78.2%	2.06 [1.18, 3.60]
**Total (95% CI)**			**100.0%**	**2.22 [1.36, 3.63]**

Heterogeneity: Chi² = 0.32, df = 1 (P = 0.57); I² = 0%
Test for overall effect: Z = 3.17 (P = 0.002)

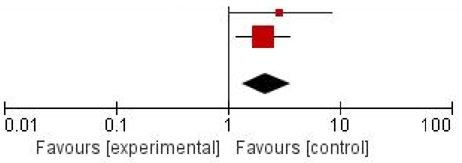

Operation time

Freire,2015(CRAB)	0.0392	0.0149	55.7%	1.04 [1.01, 1.07]
Freire,2017	-0.5212	0.189	44.3%	0.59 [0.41, 0.86]
**Total (95% CI)**			**100.0%**	**0.81 [0.47, 1.40]**

Heterogeneity: Tau² = 0.14; Chi² = 8.74, df = 1 (P = 0.003); I² = 89%
Test for overall effect: Z = 0.75 (P = 0.45)

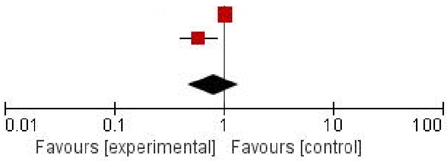

Renal replacement therapy

Francesco,2016	2.4678	0.6848	15.6%	11.80 [3.08, 45.15]
Freire,2017	1.6827	0.2946	84.4%	5.38 [3.02, 9.58]
**Total (95% CI)**			**100.0%**	**6.08 [3.58, 10.34]**

Heterogeneity: Chi² = 1.11, df = 1 (P = 0.29); I² = 10%
Test for overall effect: Z = 6.67 (P < 0.00001)

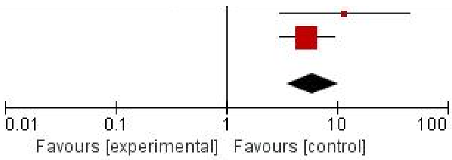

Supplement: Supplemental Material [file IANN_A_2314236_SM1791.zip › suppl_data/Figure S2 Forest plots for risk factors of mortality.pdf]
