# Supplementary material for: A comprehensive evaluation of risk factors for mortality, infection and colonization associated with CRGNB in adult solid organ transplant recipients: a systematic review and meta-analysis
Source: Ann Med. 2024 Mar 5;56(1):2314236. doi: 10.1080/07853890.2024.2314236 (PMC10916923; doi:10.1080/07853890.2024.2314236)

Study or Subgroup	log[Risk Ratio]	SE	Weight	Risk Ratio IV, Fixed, 95% CI
**Reoperation**				
LT				
Cinar,2019	0.6043	0.569	11.7%	1.83 [0.60, 5.58]
Freire,2015(CRAB)	1.1663	0.5063	14.7%	3.21 [1.19, 8.66]
Freire,2017	0.4121	0.2364	67.6%	1.51 [0.95, 2.40]
**Subtotal (95% CI)**			**94.0%**	**1.74 [1.18, 2.58]**
Heterogeneity: Chi² = 1.83, df = 2 (P = 0.40); I² = 0%				
Test for overall effect: Z = 2.76 (P = 0.006)				
KT				
Zhang,2021	1.0357	0.7968	6.0%	2.82 [0.59, 13.43]
**Subtotal (95% CI)**			**6.0%**	**2.82 [0.59, 13.43]**
Heterogeneity: Not applicable				
Test for overall effect: Z = 1.30 (P = 0.19)				
**Total (95% CI)**			**100.0%**	**1.79 [1.22, 2.62]**
Heterogeneity: Chi² = 2.17, df = 3 (P = 0.54); I² = 0%				
Test for overall effect: Z = 3.00 (P = 0.003)				
Test for subgroup differences: Chi² = 0.34, df = 1 (P = 0.56), I² = 0%				

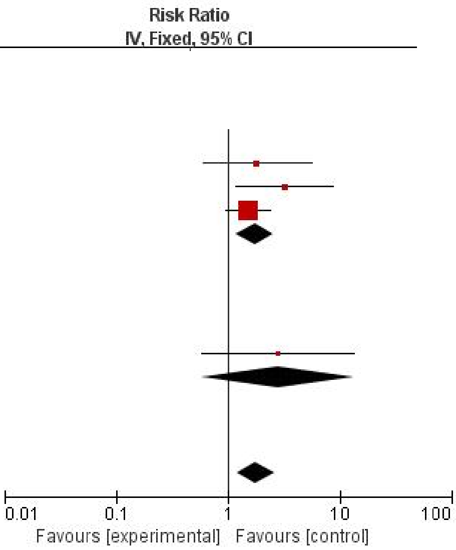

Study or Subgroup	log[Risk Ratio]	SE	Weight	Risk Ratio IV, Fixed, 95% CI
CRE				
Cinar,2019	0.6043	0.569	11.7%	1.83 [0.60, 5.58]
Freire,2017	0.4121	0.2364	67.6%	1.51 [0.95, 2.40]
**Subtotal (95% CI)**			**79.3%**	**1.55 [1.01, 2.38]**
Heterogeneity: Chi² = 0.10, df = 1 (P = 0.76); I² = 0%				
Test for overall effect: Z = 2.02 (P = 0.04)				
CRKP				
Zhang,2021	1.0357	0.7968	6.0%	2.82 [0.59, 13.43]
**Subtotal (95% CI)**			**6.0%**	**2.82 [0.59, 13.43]**
Heterogeneity: Not applicable				
Test for overall effect: Z = 1.30 (P = 0.19)				
CRAB				
Freire,2015(CRAB)	1.1663	0.5063	14.7%	3.21 [1.19, 8.66]
**Subtotal (95% CI)**			**14.7%**	**3.21 [1.19, 8.66]**
Heterogeneity: Not applicable				
Test for overall effect: Z = 2.30 (P = 0.02)				
**Total (95% CI)**			**100.0%**	**1.79 [1.22, 2.62]**
Heterogeneity: Chi² = 2.17, df = 3 (P = 0.54); I² = 0%				
Test for overall effect: Z = 3.00 (P = 0.003)				
Test for subgroup differences: Chi² = 2.08, df = 2 (P = 0.35), I² = 3.7%				

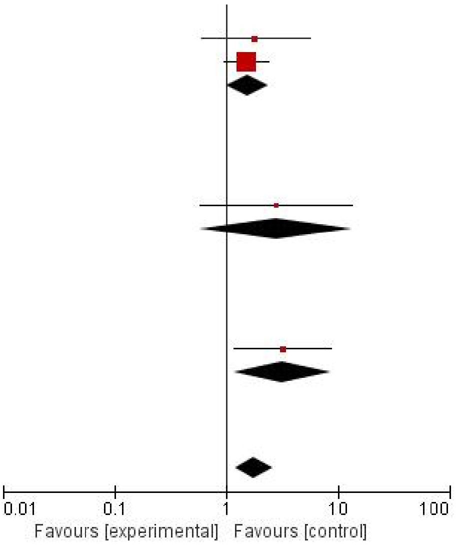

Posttransplantation infections

Study or Subgroup	log[Risk Ratio]	SE	Weight	Risk Ratio IV, Fixed, 95% CI
LT				
Cinar,2019	1.805	3.0989	0.4%	6.08 [0.01, 2640.52]
Francesco,2016	1.716	0.7885	6.5%	5.56 [1.19, 26.09]
Freire,2015(CRAB)	0.9478	0.3497	32.9%	2.58 [1.30, 5.12]
Kalpoe,2012	1.5892	0.604	11.0%	4.90 [1.50, 16.01]
Pereira,2016	1.9344	0.3872	26.8%	6.92 [3.24, 14.78]
**Subtotal (95% CI)**			**77.6%**	**4.26 [2.72, 6.65]**
Heterogeneity: Chi² = 3.81, df = 4 (P = 0.43); I² = 0%				
Test for overall effect: Z = 6.36 (P < 0.00001)				
SOT				
Nicole,2021	0.9207	0.5785	12.0%	2.51 [0.81, 7.80]
Wu,2020	3.0926	0.828	5.9%	22.03 [4.35, 111.66]
**Subtotal (95% CI)**			**17.9%**	**5.12 [2.02, 12.97]**
Heterogeneity: Chi² = 4.62, df = 1 (P = 0.03); I² = 78%				
Test for overall effect: Z = 3.44 (P = 0.0006)				
KT				
Zhang,2021	1.9097	0.949	4.5%	6.75 [1.05, 43.37]
**Subtotal (95% CI)**			**4.5%**	**6.75 [1.05, 43.37]**
Heterogeneity: Not applicable				
Test for overall effect: Z = 2.01 (P = 0.04)				
**Total (95% CI)**			**100.0%**	**4.49 [3.03, 6.65]**
Heterogeneity: Chi² = 8.75, df = 7 (P = 0.27); I² = 20%				
Test for overall effect: Z = 7.49 (P < 0.00001)				
Test for subgroup differences: Chi² = 0.32, df = 2 (P = 0.85), I² = 0%				

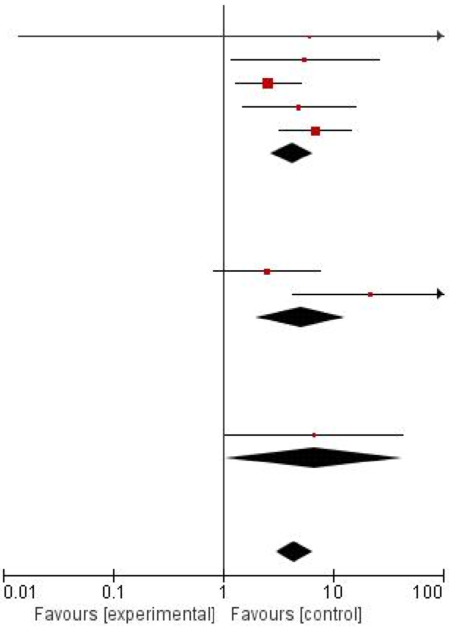

**CRKP**				
Francesco,2016	1.716	0.7885	6.5%	5.56 [1.19, 26.09]
Kalpoe,2012	1.5892	0.604	11.0%	4.90 [1.50, 16.01]
Nicole,2021	0.9207	0.5785	12.0%	2.51 [0.81, 7.80]
Pereira,2016	1.9344	0.3872	26.8%	6.92 [3.24, 14.78]
Wu,2020	3.0926	0.828	5.9%	22.03 [4.35, 111.66]
Zhang,2021	1.9097	0.949	4.5%	6.75 [1.05, 43.37]
**Subtotal (95% CI)**			**66.7%**	**5.89 [3.64, 9.54]**
Heterogeneity: Chi² = 5.00, df = 5 (P = 0.42); I² = 0%				
Test for overall effect: Z = 7.22 (P < 0.00001)				
**CRE**				
Cinar,2019	1.805	3.0989	0.4%	6.08 [0.01, 2640.52]
**Subtotal (95% CI)**			**0.4%**	**6.08 [0.01, 2640.52]**
Heterogeneity: Not applicable				
Test for overall effect: Z = 0.58 (P = 0.56)				
**CRAB**				
Freire,2015(CRAB)	0.9478	0.3497	32.9%	2.58 [1.30, 5.12]
**Subtotal (95% CI)**			**32.9%**	**2.58 [1.30, 5.12]**
Heterogeneity: Not applicable				
Test for overall effect: Z = 2.71 (P = 0.007)				
**Total (95% CI)**			**100.0%**	**4.49 [3.03, 6.65]**
Heterogeneity: Chi² = 8.75, df = 7 (P = 0.27); I² = 20%				
Test for overall effect: Z = 7.49 (P < 0.00001)				
Test for subgroup differences: Chi² = 3.74, df = 2 (P = 0.15), I² = 46.6%				

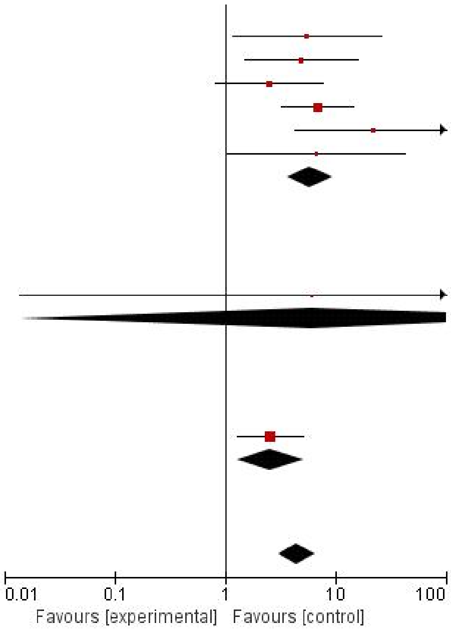

Supplement: Supplemental Material [file IANN_A_2314236_SM1791.zip › suppl_data/Figure S3 Subgroup analyses of risk factors for mortality.pdf]
