# Supplementary material for: A comprehensive evaluation of risk factors for mortality, infection and colonization associated with CRGNB in adult solid organ transplant recipients: a systematic review and meta-analysis
Source: Ann Med. 2024 Mar 5;56(1):2314236. doi: 10.1080/07853890.2024.2314236 (PMC10916923; doi:10.1080/07853890.2024.2314236)

Study or Subgroup	log[Odds Ratio]	SE	Weight	Odds Ratio IV, Fixed, 95% CI
**Prolonged mechanical Ventilation>48h**				
Chen ,2020	0.8519	0.6655	11.2%	2.34 [0.64, 8.64]
Cinar,2019	0.6627	1.0454	4.6%	1.94 [0.25, 15.05]
Giannella,2015	1.7475	0.5805	14.8%	5.74 [1.84, 17.91]
Giannella,2019	0.967	0.2933	57.9%	2.63 [1.48, 4.67]
Zhang,2021	1.3135	0.658	11.5%	3.72 [1.02, 13.51]
**Total (95% CI)**			**100.0%**	**2.99 [1.93, 4.63]**

Heterogeneity: Chi² = 1.87, df = 4 (P = 0.76); I² = 0%
Test for overall effect: Z = 4.91 (P < 0.00001)

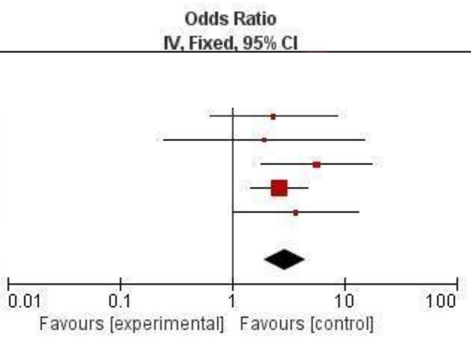

Study or Subgroup	log[Odds Ratio]	SE	Weight	Odds Ratio IV, Fixed, 95% CI
**Combined transplantation**				
Freire,2015(CRKP)	1.7509	0.5004	24.9%	5.76 [2.16, 15.36]
Freire,2017	1.1249	0.574	18.9%	3.08 [1.00, 9.49]
Freire,2021（CRE）	1.3083	0.6189	16.3%	3.70 [1.10, 12.45]
Giannella,2019	0.9555	0.3945	40.0%	2.60 [1.20, 5.63]
**Total (95% CI)**			**100.0%**	**3.46 [2.12, 5.65]**

Heterogeneity: Chi² = 1.61, df = 3 (P = 0.66); I² = 0%
Test for overall effect: Z = 4.98 (P < 0.00001)

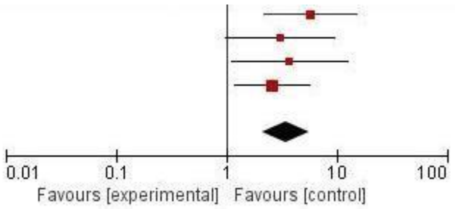

Study or Subgroup	log[Odds Ratio]	SE	Weight	Odds Ratio IV, Fixed, 95% CI
**Reoperation**				
Chen ,2020	0.9832	0.633	9.9%	2.67 [0.77, 9.24]
Freire,2017	0.8242	0.4005	24.8%	2.28 [1.04, 5.00]
Giannella,2019	0.7701	0.2957	45.4%	2.16 [1.21, 3.86]
Nguyen,2021	2.3224	0.9451	4.4%	10.20 [1.60, 65.02]
Zhang,2021	-0.102	0.5074	15.4%	0.90 [0.33, 2.44]
**Total (95% CI)**			**100.0%**	**2.09 [1.42, 3.09]**

Heterogeneity: Chi² = 5.76, df = 4 (P = 0.22); I² = 31%
Test for overall effect: Z = 3.71 (P = 0.0002)

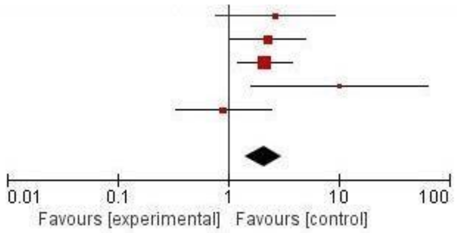

Study or Subgroup	log[Odds Ratio]	SE	Weight	Odds Ratio IV, Fixed, 95% CI
**Pre-transplantation CRGNB colonization**				
Freire,2015(CRAB)	0.9439	0.5132	21.6%	2.57 [0.94, 7.03]
Freire,2017	2.5455	0.4207	23.7%	12.75 [5.59, 29.08]
Giannella,2015	2.8124	0.5717	20.3%	16.65 [5.43, 51.06]
Giannella,2019	2.9178	0.5137	21.6%	18.50 [6.76, 50.63]
Nguyen,2021	4.3135	0.9774	12.7%	74.70 [11.00, 507.33]
**Total (95% CI)**			**100.0%**	**12.91 [5.23, 31.88]**

Heterogeneity: Tau² = 0.72; Chi² = 13.52, df = 4 (P = 0.009); I² = 70%
Test for overall effect: Z = 5.55 (P < 0.00001)

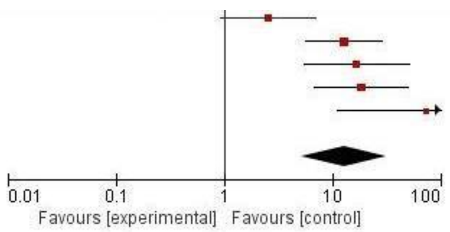

Study or Subgroup	log[Odds Ratio]	SE	Weight	Odds Ratio IV, Fixed, 95% CI
**Delayed colonization function**				
Taminato,2019	1.7228	0.5488	37.4%	5.60 [1.91, 16.42]
Varotti,2017	0.088	0.9673	21.8%	1.09 [0.16, 7.27]
Zhang,2021	0.179	0.4787	40.7%	1.20 [0.47, 3.06]
**Total (95% CI)**			**100.0%**	**2.09 [0.68, 6.46]**

Heterogeneity: Tau² = 0.58; Chi² = 5.03, df = 2 (P = 0.08); I² = 60%
Test for overall effect: Z = 1.28 (P = 0.20)

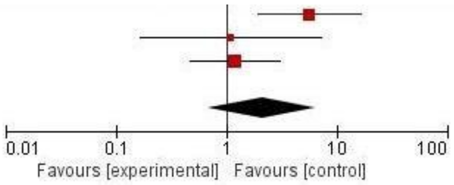

Study or Subgroup	log[Odds Ratio]	SE	Weight	Odds Ratio IV, Fixed, 95% CI
**Mean length of post-transplantation ICU stay**				
Cinar,2019	-0.2877	0.3207	0.1%	0.75 [0.40, 1.41]
Kim,2018	0.1221	0.009	99.9%	1.13 [1.11, 1.15]
**Total (95% CI)**			**100.0%**	**1.13 [1.11, 1.15]**

Heterogeneity: Chi² = 1.63, df = 1 (P = 0.20); I² = 39%
Test for overall effect: Z = 13.54 (P < 0.00001)

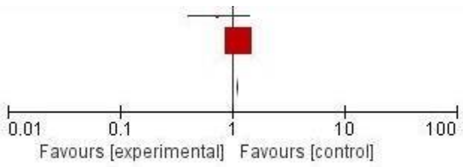

Renal replacement therapy

Study or Subgroup	log[Odds Ratio]	SE	Weight	Odds Ratio IV, Fixed, 95% CI
Chen ,2020	1.325	0.5847	11.3%	3.76 [1.20, 11.83]
Freire,2015(CRAB)	1.1249	0.3809	26.7%	3.08 [1.46, 6.50]
Freire,2017	0.7227	0.3487	31.9%	2.06 [1.04, 4.08]
Giannella,2015	1.5581	0.5426	13.2%	4.75 [1.64, 13.76]
Kim,2018	0.2231	0.9888	4.0%	1.25 [0.18, 8.68]
Taminato,2019	1.7228	0.5488	12.9%	5.60 [1.91, 16.42]
**Total (95% CI)**			**100.0%**	**3.06 [2.08, 4.50]**

Heterogeneity: Chi² = 4.10, df = 5 (P = 0.53); I² = 0%
Test for overall effect: Z = 5.67 (P < 0.00001)

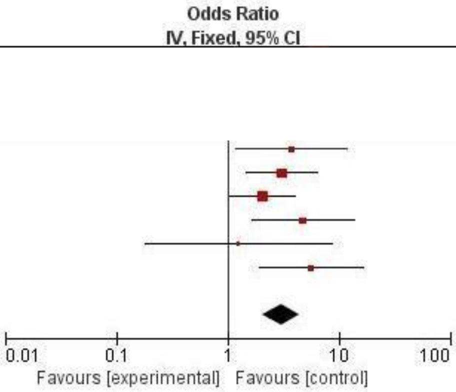

Post-LT CRGNB colonization Pre-LT liver disease

Study or Subgroup	log[Odds Ratio]	SE	Weight	Odds Ratio IV, Fixed, 95% CI
Chen ,2020	1.7077	0.4896	24.3%	5.52 [2.11, 14.40]
Freire,2017	1.4207	0.3894	28.6%	4.14 [1.93, 8.88]
Giannella,2015	2.8124	0.5717	21.2%	16.65 [5.43, 51.06]
Giannella,2019	2.8267	0.4531	25.8%	16.89 [6.95, 41.05]
**Total (95% CI)**			**100.0%**	**8.58 [4.08, 18.04]**

Heterogeneity: Tau² = 0.35; Chi² = 7.80, df = 3 (P = 0.05); I² = 62%
Test for overall effect: Z = 5.67 (P < 0.00001)

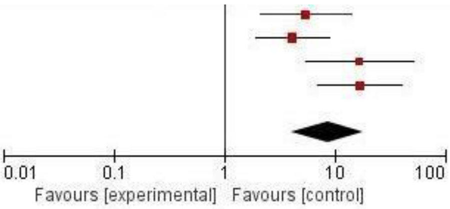

Pre-LT liver disease

Study or Subgroup	log[Odds Ratio]	SE	Weight	Odds Ratio IV, Fixed, 95% CI
Chen ,2020	1.4095	0.7673	15.4%	4.09 [0.91, 18.42]
Freire,2015(CRAB)	1.2267	0.5371	31.4%	3.41 [1.19, 9.77]
Giannella,2015	2.2721	0.7084	18.0%	9.70 [2.42, 38.88]
Pereira,2016	1.16	0.5074	35.2%	3.19 [1.18, 8.62]
**Total (95% CI)**			**100.0%**	**4.14 [2.29, 7.46]**

Heterogeneity: Chi² = 1.84, df = 3 (P = 0.61); I² = 0%
Test for overall effect: Z = 4.72 (P < 0.00001)

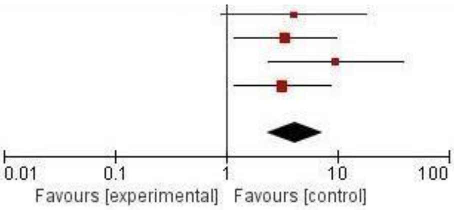

MELD score

Study or Subgroup	log[Odds Ratio]	SE	Weight	Odds Ratio IV, Fixed, 95% CI
Chen ,2020	0.006	0.0292	15.1%	1.01 [0.95, 1.07]
Cinar,2019	0.5988	2.0946	0.0%	1.82 [0.03, 110.40]
Freire,2017	0.7227	0.3637	0.1%	2.06 [1.01, 4.20]
Giannella,2019	0.0296	0.0151	56.6%	1.03 [1.00, 1.06]
Kim,2018	0.0677	0.0448	6.4%	1.07 [0.98, 1.17]
Pereira,2016	0.0677	0.0244	21.7%	1.07 [1.02, 1.12]
**Total (95% CI)**			**100.0%**	**1.04 [1.02, 1.06]**

Heterogeneity: Chi² = 7.04, df = 5 (P = 0.22); I² = 29%
Test for overall effect: Z = 3.29 (P = 0.0010)

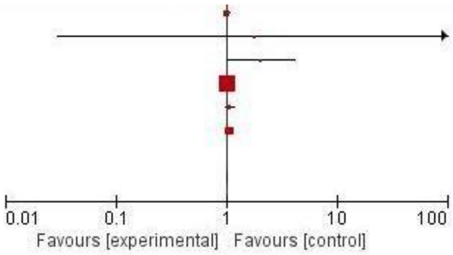

Biliary complication

Study or Subgroup	log[Odds Ratio]	SE	Weight	Odds Ratio IV, Fixed, 95% CI
Chen ,2020	1.3295	0.6617	40.5%	3.78 [1.03, 13.82]
Pereira,2016	1.7733	0.546	59.5%	5.89 [2.02, 17.17]
**Total (95% CI)**			**100.0%**	**4.92 [2.16, 11.23]**

Heterogeneity: Chi² = 0.27, df = 1 (P = 0.60); I² = 0%
Test for overall effect: Z = 3.78 (P = 0.0002)

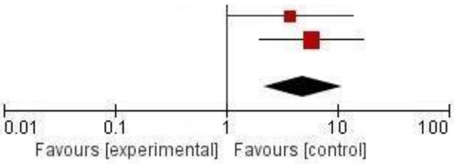

Age

Study or Subgroup	log[Odds Ratio]	SE	Weight	Odds Ratio IV, Fixed, 95% CI
Freire,2015(CRKP)	0.0862	0.044	20.4%	1.09 [1.00, 1.19]
Zhang,2021	0.0296	0.0223	79.6%	1.03 [0.99, 1.08]
**Total (95% CI)**			**100.0%**	**1.04 [1.00, 1.08]**

Heterogeneity: Chi² = 1.32, df = 1 (P = 0.25); I² = 24%
Test for overall effect: Z = 2.07 (P = 0.04)

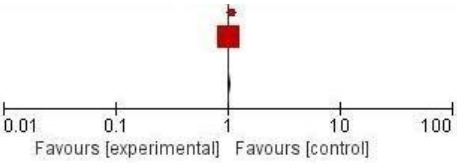

Supplement: Supplemental Material [file IANN_A_2314236_SM1791.zip › suppl_data/Figure S4 Forest plots for risk factors of infection.PDF]
