# Supplementary material for: A comprehensive evaluation of risk factors for mortality, infection and colonization associated with CRGNB in adult solid organ transplant recipients: a systematic review and meta-analysis
Source: Ann Med. 2024 Mar 5;56(1):2314236. doi: 10.1080/07853890.2024.2314236 (PMC10916923; doi:10.1080/07853890.2024.2314236)
Supplement: Supplemental Material [file IANN_A_2314236_SM1791.zip › suppl_data/Table S1.DOCX]

**Table S1. Quality assessment according to the Newcastle-Ottawa Scale for the included studies.**

**Case-control study NOS score**

| **Study** | **Selection** | | | | **Comparability** | **Exposure** | | | **Total score** |
| --- | --- | --- | --- | --- | --- | --- | --- | --- | --- |
|  | **Is the case definition adequate?** | **Representativeness of the cases** | **Selection of Controls** | **Definition of Controls** | **Comparability of cases and controls on the basis of the design or analysis** | **Ascertainment of exposure** | **Same method of ascertainment for cases and controls** | **Non-Response rate** |  |
| Chen,2020^15^ | * | * | * | * | * | * | * | * | 8 |
| Freire, 2016^8^ | * | * | * | * | * | * | * | * | 8 |
| Cinar, 2019^17^ | * | * |  | * | * | * | * | * | 7 |
| Freire,2021^12^（CRE） | * | * | * | * | * | * | * | * | 8 |
| Kim,2018^18^ | * | * |  | * | * | * | * | * | 7 |
| Freire,2021^9^（CRPA） | * | * | * | * | * | * | * | * | 8 |
| Lee,2018^19^ | * | * |  | * | * | * | * | * | 7 |
| Zhang,2021^16^ | * | * | * | * | ** | * | * | * | 9 |
| Taminato,2019^14^ | * | * |  | * | ** | * | * | * | 8 |
| Mazza,2017^21^ | * | * |  | * | * | * | * | * | 7 |
| Varotti, 2017^23^ | * | * |  | * | * | * | * | * | 7 |

**Cohort study NOS score**

| **Study** | **Selection** | | | | **Comparability** | Outcome | | | **Total score** |
| --- | --- | --- | --- | --- | --- | --- | --- | --- | --- |
|  | **Representativeness of the exposed cohort** | **Selection of the non exposed cohort** | **Ascertainment of exposure** | **Demonstration that outcome of interest was not present at start of study** | **Comparability of cohorts on the basis of the design or analysis** | **Assessment of outcome** | **Was follow-up long enough for outcomes to occur** | **Adequacy of follow up of cohorts** |  |
| Giannella,2019^20^ | * | * | * | * | * | * | * | * | 8 |
| Freire,2017^13^ | * | * | * |  | * | * | * | * | 7 |
| Freire,2015^11^（CRKP） | * | * | * |  | * | * | * | * | 7 |
| Nguyen, 2021^24^ | * | * | * | * | * | * | * | * | 8 |
| Freire,2015^10^（CRAB） | * | * | * |  | * | * | * | * | 7 |
| Kalpoe,2012^25^ | * | * | * | * | * | * | * | * | 8 |
| Pereira,2016^26^ | * | * | * |  | * | * | * | * | 7 |
| Giannella,2015^22^ | * | * | * | * | * | * | * | * | 8 |
