# Supplementary material for: A comprehensive evaluation of risk factors for mortality, infection and colonization associated with CRGNB in adult solid organ transplant recipients: a systematic review and meta-analysis
Source: Ann Med. 2024 Mar 5;56(1):2314236. doi: 10.1080/07853890.2024.2314236 (PMC10916923; doi:10.1080/07853890.2024.2314236)
Supplement: Supplemental Material [file IANN_A_2314236_SM1791.zip › suppl_data/Table S2 Risk factors for mortality in SOT recipients.docx]

**Table S2. Risk factors for mortality in SOT recipients.**

|  | **Number of**  **studies** | **Heterogeneity** | | | **RR [95%CI]** | **Z** | ***P*** | **Effect**  **model** | **Egger’s test, *P*>\|t\|** | **Begg’s test** |
| --- | --- | --- | --- | --- | --- | --- | --- | --- | --- | --- |
|  |  | **χ^2^** | ***P*** | ***I^2^*** |  |  |  |  |  |  |
| **SOT** |  |  |  |  |  |  |  |  |  |  |
| Reoperation | 4 | 2.17 | 0.54 | 0% | 1.79 [1.22, 2.62] | 0 | 0.003 | Fixed | 0.317 | 1 |
| Post-transplantation infections | 8 | 8.75 | 0.27 | 20% | 4.49 [3.03, 6.65] | 7.49 | <0.00001 | Fixed | 0.314 | 0.266 |
| Delayed graft function | 2 | 1.52 | 0.22 | 34% | 4.89 [2.70, 8.86] | 5.24 | <0.00001 | Fixed | NA | 1 |
| Mechanical ventilation | 2 | 0.35 | 0.55 | 0% | 8.59 [3.55, 20.79] | 4.77 | <0.00001 | Fixed | NA | 1 |
| Septic shock | 2 | 0.79 | 0.37 | 0% | 8.56 [3.49, 21.00] | 4.69 | <0.00001 | Fixed | NA | 1 |
| **LT** |  |  |  |  |  |  |  |  |  |  |
| Fulminant hepatitis | 2 | 0.32 | 0.57 | 0% | 2.22 [1.36, 3.63] | 3.17 | 0.002 | Fixed | NA | 1 |
| Operation time | 2 | 8.74 | 0.003 | 89% | 0.81 [0.47, 1.40] | 0.75 | 0.45 | Random | NA | 1 |
| Renal replacement therapy | 2 | 1.11 | 0.29 | 10% | 6.08 [3.58, 10.34] | 6.67 | <0.00001 | Fixed | NA | 1 |
