# Supplementary material for: A comprehensive evaluation of risk factors for mortality, infection and colonization associated with CRGNB in adult solid organ transplant recipients: a systematic review and meta-analysis
Source: Ann Med. 2024 Mar 5;56(1):2314236. doi: 10.1080/07853890.2024.2314236 (PMC10916923; doi:10.1080/07853890.2024.2314236)
Supplement: Supplemental Material [file IANN_A_2314236_SM1791.zip › suppl_data/Table S3.DOCX]

**Table S3. Literature related to CRGNB colonization before LT**

**transplantation.**

| **Study** | **Pretransplant CRGNB acquisition, n** | **Number of post-transplant infections, n** | **Time to infection after transplant** |
| --- | --- | --- | --- |
|  |  |  |  |
| Giannella,2019^20^ | 38 | 14 | 3~21.5d, Median time 13d |
| Freire, 2017^13^ | 57 | 29 | 0-219d, Median time 8 d |
| Freire, 2015^10^（CRAB） | 24 | 11 | NA |
| Nguyen, 2021^24^ | 10 | 8 | NA |
| Giannella, 2015^22^ | 11 | 2 | 10d and 131d |
