# Supplementary material for: A comprehensive evaluation of risk factors for mortality, infection and colonization associated with CRGNB in adult solid organ transplant recipients: a systematic review and meta-analysis
Source: Ann Med. 2024 Mar 5;56(1):2314236. doi: 10.1080/07853890.2024.2314236 (PMC10916923; doi:10.1080/07853890.2024.2314236)
Supplement: Supplemental Material [file IANN_A_2314236_SM1791.zip › suppl_data/Table S4.DOCX]

**Table S4. Literature related to CRGNB colonization after LT**

| **Study** | **post-transplant CRGNB acquisition, n** | **Number of post-transplant infections, n** | **Time to infection after transplant** |
| --- | --- | --- | --- |
|  |  |  |  |
| Giannella, 2015^22^ | 30 | 14 | Median time 40d |
| Freire, 2017^13^ | 104 | 27 | 0-219d, Median time 8 d |
| Giannella,2019^20^ | 109 | 37 | 14-129d, Median time 37d |
| Chen, 2020^15^ | 65 | 13 | NA |

**transplantation.**
