# Supplementary figures and images for: A comprehensive evaluation of risk factors for mortality, infection and colonization associated with CRGNB in adult solid organ transplant recipients: a systematic review and meta-analysis
Source: Ann Med. 2024 Mar 5;56(1):2314236. doi: 10.1080/07853890.2024.2314236 (PMC10916923; doi:10.1080/07853890.2024.2314236)

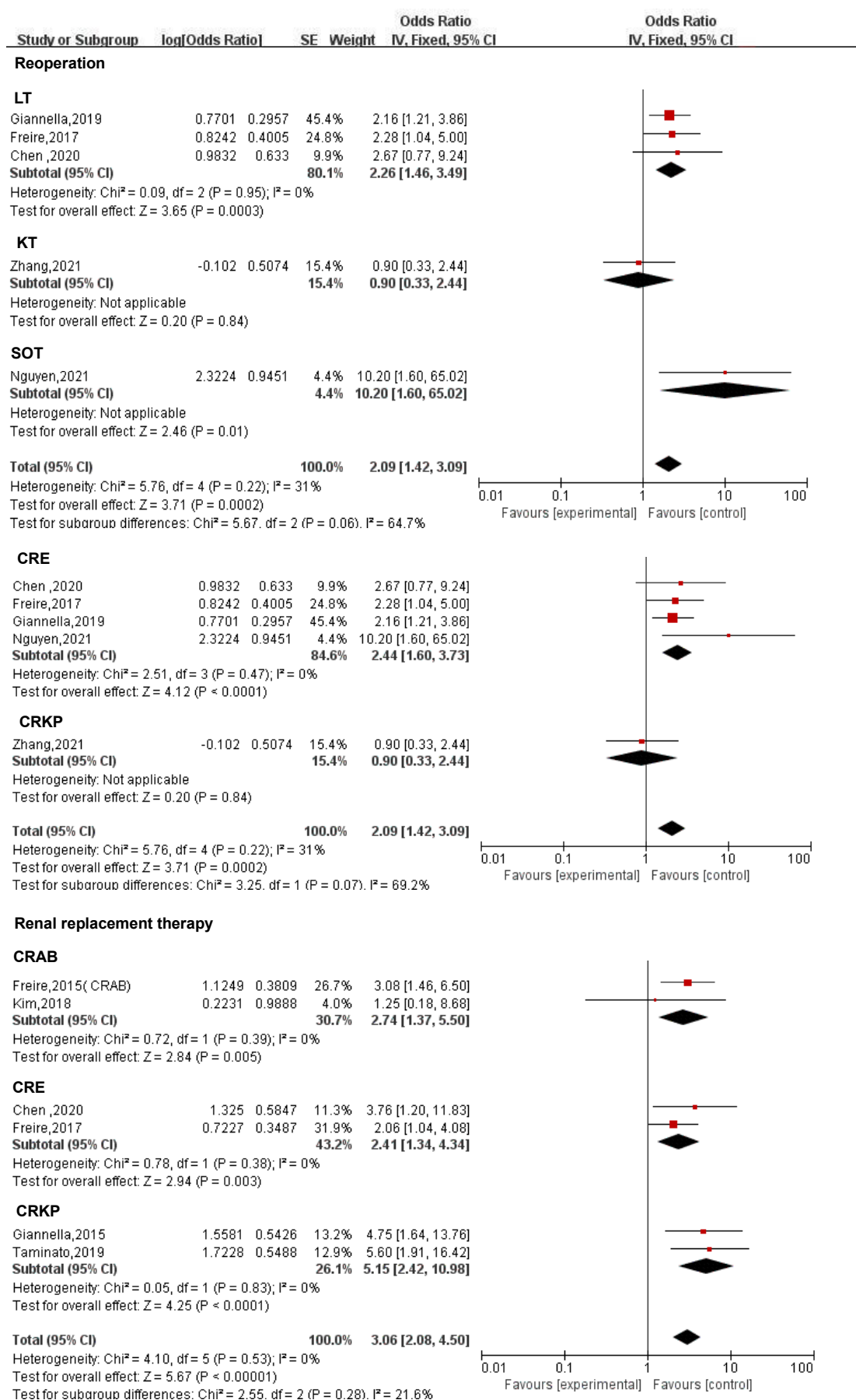

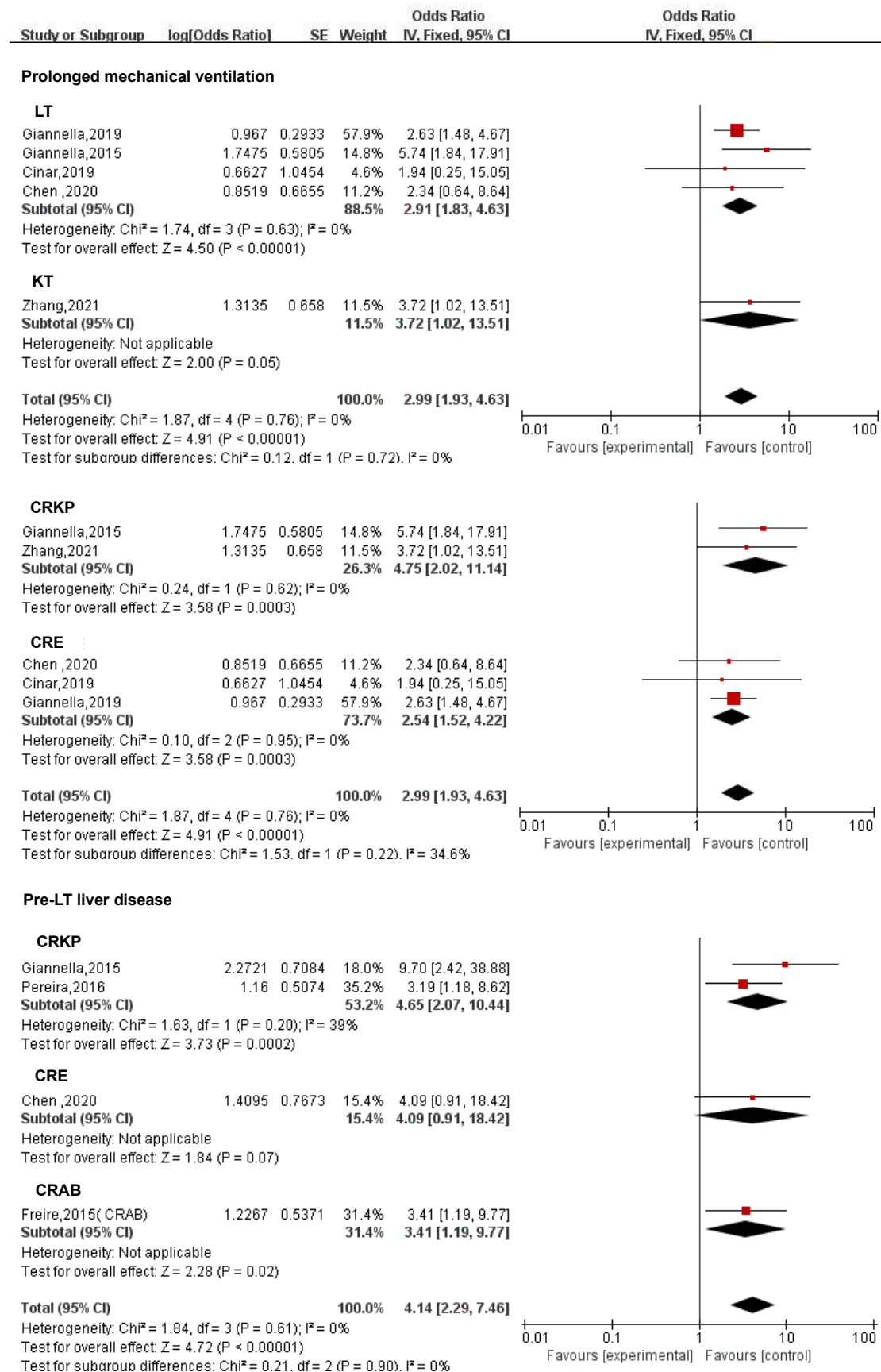

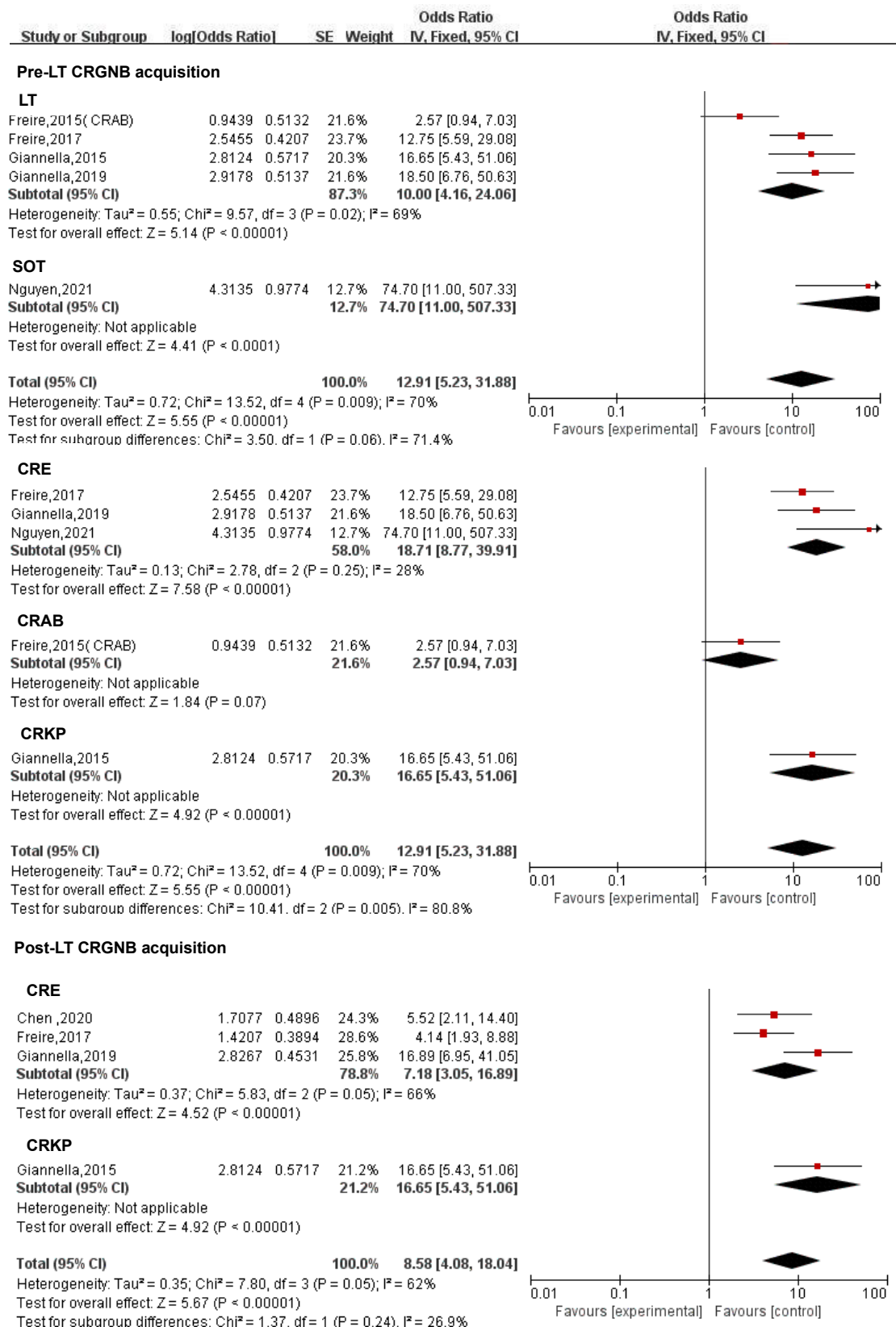

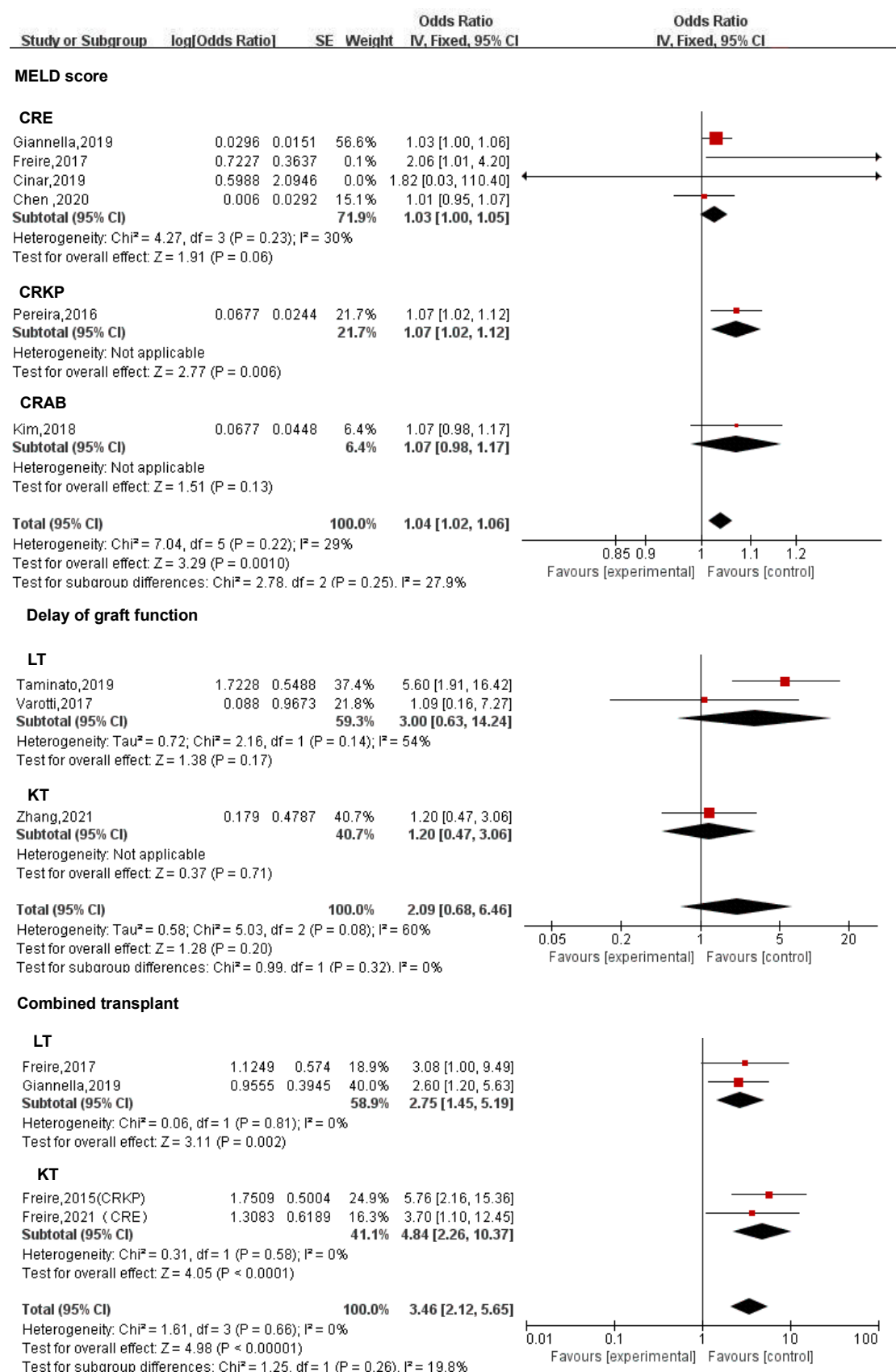

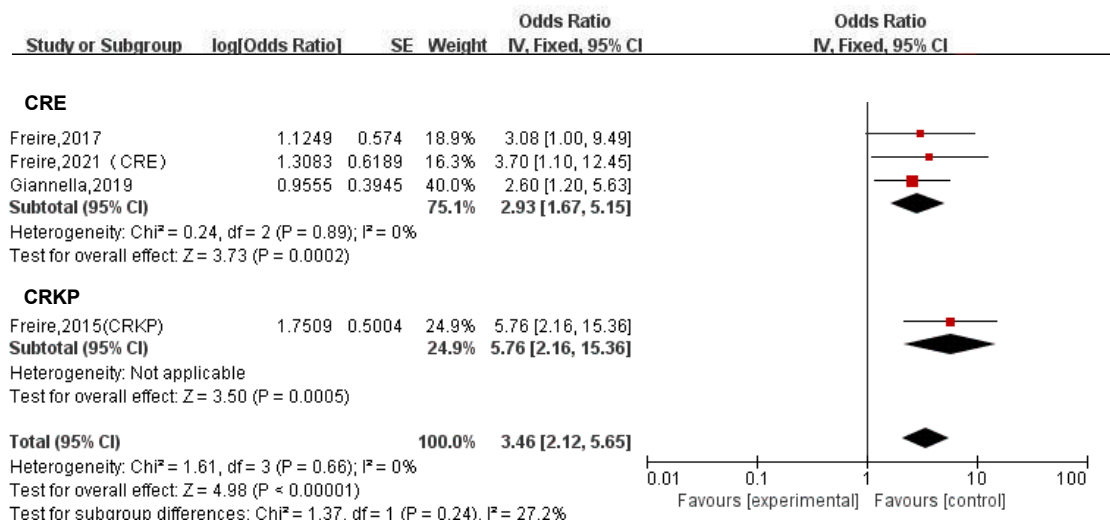

Supplement: Supplemental Material [file IANN_A_2314236_SM1791.zip › suppl_data/Figure S5 Subgroup analyses of risk factors for infection.PDF]

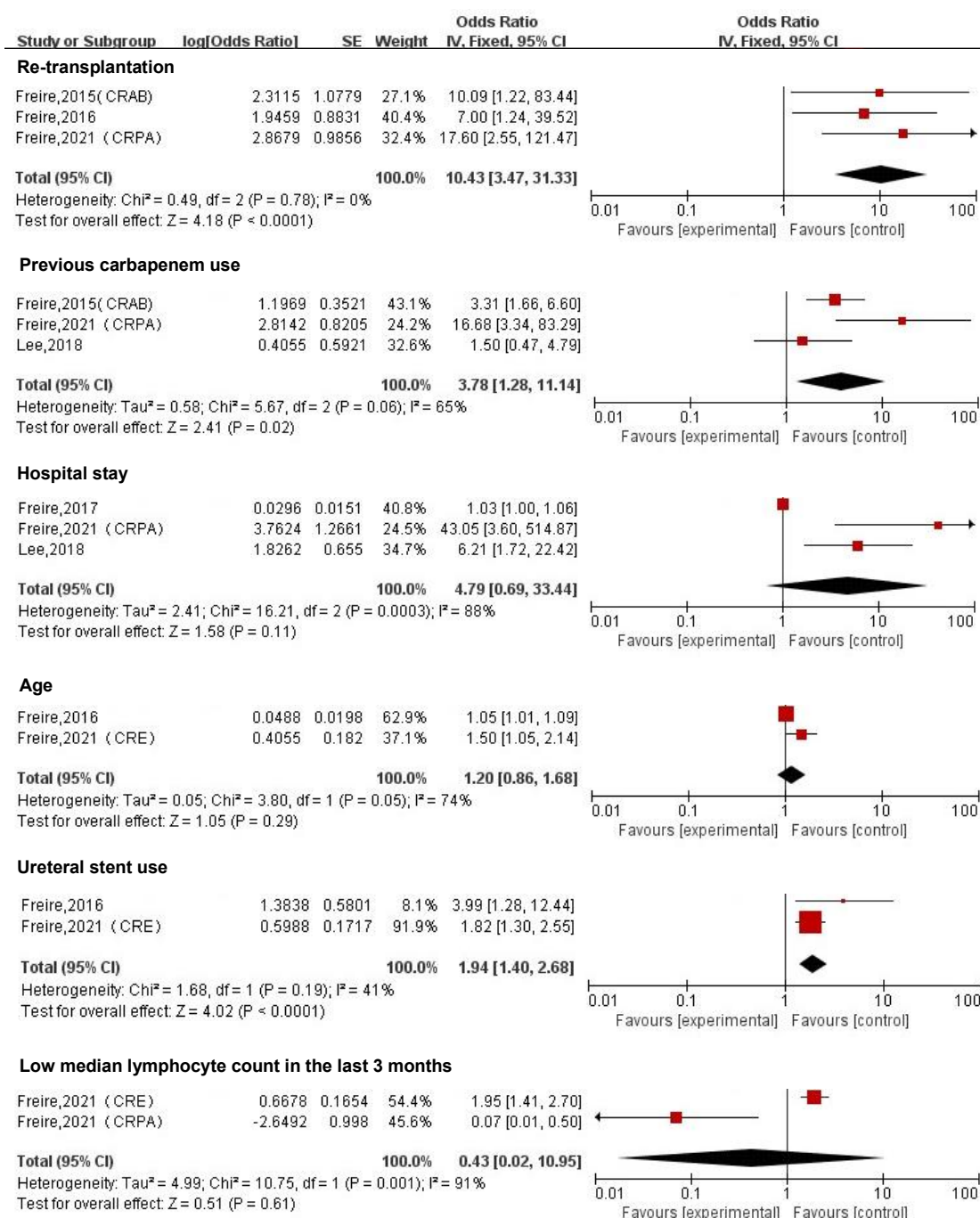

Supplement: Supplemental Material [file IANN_A_2314236_SM1791.zip › suppl_data/Figure S6 Forest plots for risk factors of colonization.PDF]

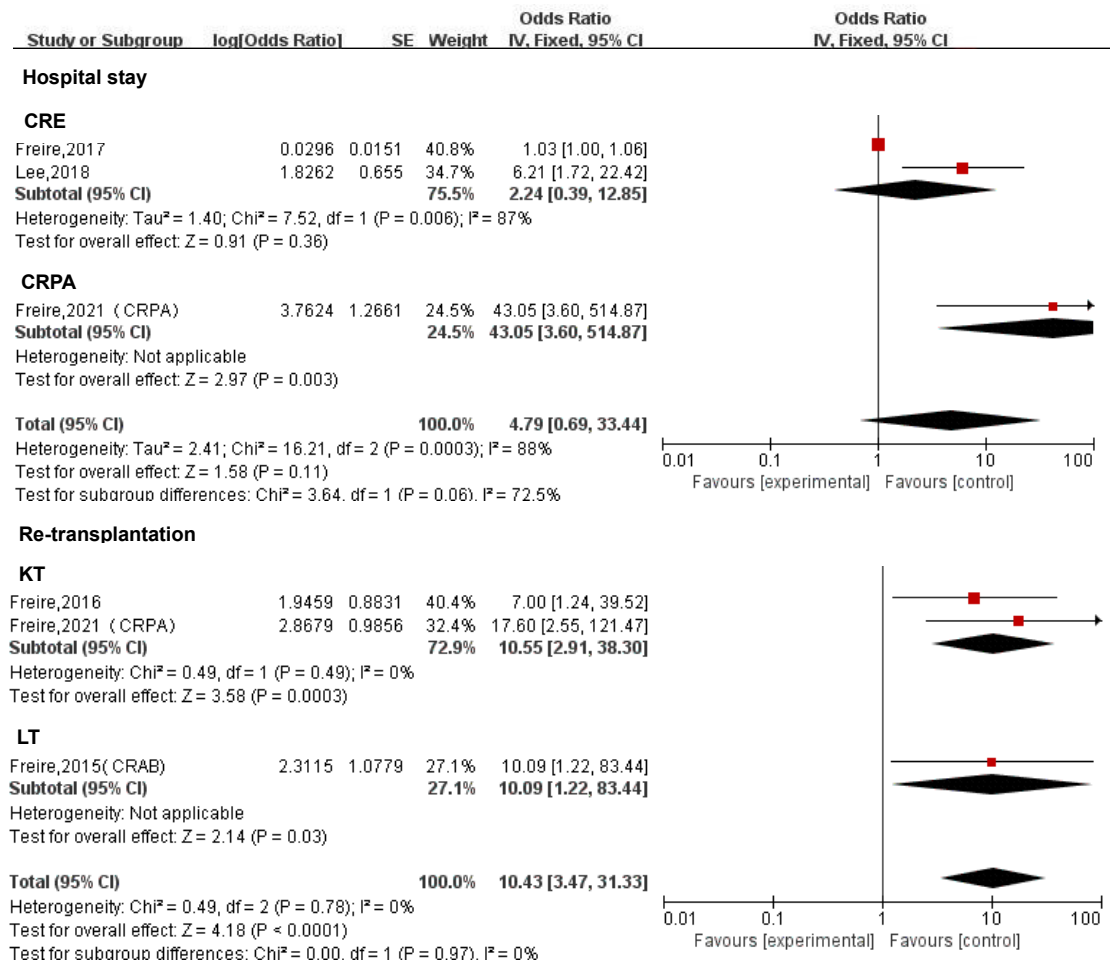

Supplement: Supplemental Material [file IANN_A_2314236_SM1791.zip › suppl_data/Figure S7 Subgroup analyses of risk factors for colonization.PDF]
